# The TOR Signaling Pathway in Spatial and Temporal Control of Cell Size and Growth

**DOI:** 10.3389/fcell.2017.00061

**Published:** 2017-06-07

**Authors:** Suam Gonzalez, Charalampos Rallis

**Affiliations:** School of Health, Sport and Bioscience, University of East LondonLondon, United Kingdom

**Keywords:** cell size, cell cycle, growth, rapamycin, signaling, nutrients, TORC1, TORC2

## Abstract

Cell size is amenable by genetic and environmental factors. The highly conserved nutrient-responsive Target of Rapamycin (TOR) signaling pathway regulates cellular metabolic status and growth in response to numerous inputs. Timing and duration of TOR pathway activity is pivotal for both cell mass built up as well as cell cycle progression and is controlled and fine-tuned by the abundance and quality of nutrients, hormonal signals, growth factors, stress, and oxygen. TOR kinases function within two functionally and structurally discrete multiprotein complexes, TORC1 and TORC2, that are implicated in temporal and spatial control of cell size and growth respectively; however, recent data indicate that such functional distinctions are much more complex. Here, we briefly review roles of the two complexes in cellular growth and cytoarchitecture in various experimental model systems.

## Introduction

The Target of Rapamycin (TOR) is an evolutionarily conserved Ser/Thr-protein kinase functioning as the heart of signaling networks toward nutrient and hormonal sensing. The role of these networks is to regulate anabolism and catabolism by coordinating numerous cellular metabolic processes, such as protein translation, formation of ribosome components, mRNA stability, autophagy, cell-cycle, transcription, and cellular architecture (Laplante and Sabatini, 2012; Rallis and Bahler, 2013; Rodland et al., 2014). *Tor* genes together with the FKBP12 homolog *fpr1* were first isolated in *Saccharomyces cerevisiae* (Heitman et al., 1991; Kunz et al., 1993) as the mediators of the toxic effects of sirolimus or rapamycin, a macrolide from *Streptomyces hygroscopicus* bacteria living within the soil on the Rapa Nui or Easter Island (Sehgal et al., 1975; Sehgal, 2003). Rapamycin exhibits broad anti-proliferative properties and is a potent anti-tumor and immunosuppressant drug (Law, 2005). Rapamycin directly binds FKBP12 and the complex then binds and inhibits the TOR kinase (Yang et al., 2013). In all eukaryotes, TOR kinases are found in two distinct protein complexes, termed TOR complex 1 (TORC1) and TOR complex 2 (TORC2) (Wullschleger et al., 2006; Laplante and Sabatini, 2012; Huang and Fingar, 2014). Both complexes are implicated in cell growth. TORC1 is generally responsible for promoting ribosome biogenesis, protein anabolism and cell proliferation (Averous and Proud, 2006; Morita et al., 2015) and repressing cell differentiation (Alvarez and Moreno, 2006). The TSC1–TSC2 (hamartin and tuberin, respectively) protein complex can repress TORC1 by affecting Rheb, a G-protein that acts as positive regulator of this complex (Huang and Manning, 2008). TORC2 can have opposing or antagonistic functions to those of TORC1 (Weisman et al., 2007; Ikai et al., 2011). It gets input from carbon sources and insulin and regulates actin cytoskeleton (De Virgilio and Loewith, 2006). Fission and budding yeasts have two TOR kinases, Tor1 and Tor2 (Otsubo and Yamamato, 2008; Weisman, 2016). Fission yeast Tor1 protein is not essential and is found to be associated with both TORC1 and TORC2 (Hartmuth and Petersen, 2009). Tor1 is required for survival in stress response, appropriate G1 arrest, gene silencing, telomere integrity and sexual development. Conversely, the essential protein Tor2 is associated with TORC1 and is pivotal for growth by positively regulating protein synthesis, metabolism and transcription (Weisman et al., 2007; Otsubo and Yamamato, 2008; Ikai et al., 2011; Weisman, 2016).

TORC complexes are hubs of huge signaling networks that govern cellular growth in space in time and are heavily implicated in pathologies, such as cancer, obesity, type 2 diabetes, and neurodegeneration (Menon and Manning, 2008; Laplante and Sabatini, 2012) as well as in lifespan control and aging (Bjedov and Partridge, 2011; Rallis et al., 2013, 2014). In this mini review, we discuss roles of TORC1 and TORC2 in temporal and spatial control of cell growth and size. TORC1 while traditionally considered to be controlling temporal cell growth is also implicated in spatial control aspects with interdependent relationships with the cytoskeleton. Likewise, new interactions of TORC2 provide further links to TORC1 and temporal cell size regulation and cell cycle progression.

## Regulation of cell size and growth aspects by TORC1

Cell size is regulated depending on nutrient availability, extracellular signals and stress (Perez-Hidalgo and Moreno, 2016). TORC1 senses diverse inputs, such as nitrogen- and carbon-containing nutrients, hormonal stimulation, various stresses, availability of energy within the cell and oxygen (De Virgilio and Loewith, 2006). Interestingly, various types of nutrients have diverse effects on the duration and strength of TORC1 activity that is reflected to the cellular phenotypes observed (De Virgilio and Loewith, 2006; Stracka et al., 2014). TORC1 boosts growth by promoting protein translation, ribosome biogenesis (Averous and Proud, 2006; Ma and Blenis, 2009), and glycolysis (Laplante and Sabatini, 2012) as well as suppressing stress responses (Lopez-Maury et al., 2008) as well as autophagy mechanisms (Ganley et al., 2009; Hosokawa et al., 2009).

TOR complex 1 (TORC1) has major contributions in temporal aspects of cell size control. For example, inhibition of TORC1 using rapamycin advances mitotic onset in fission yeast. The effect is similar to cell size reduction observed when cells sense and experience poor nitrogen sources. This is achieved through the stress MAPK pathway via the Pyp2 phosphatase (Petersen and Nurse, 2007). Interestingly, regulation of mitotic entry through the MAPK Sty1 upon rapamycin treatment involves the Tor1-containing TORC1 and not the complex that contains Tor2 (Hartmuth and Petersen, 2009). In budding yeast *S. cerevisiae*, TORC1 coordinates cell size by regulating timing of G1-S cell cycle progression. This is achieved by G1 cyclins and CDK activation as well as through destabilization of Sic1, a CDK inhibitor. When TORC1 is pharmacologically inhibited or following starvation, Sic1 is stabilized by c-terminal phosphorylation. The mechanism behind this involves endosulfines activated by the greatwall kinase. These in turn will stimulate Mpk1 and will inhibit the Cdc55 protein phosphatase 2A (Moreno-Torres et al., 2015). When ample nutrients are present, fission yeast TORC1 is highly active and inhibits Greatwall (Ppk18) protein kinase and resulting in PP2A-B55 activation. PP2A-B55 activity prevents Cdk1-Cyclin B action. Cells, therefore, increase in size during G2 before they commit to mitosis. In nutrient limitations, TORC1 activity lowers and Greatwall (Ppk18) activation results in endosulfine (Igo1) phosphorylation and PP2A-B55 inhibition. These events fully activate Cdk1·CyclinB and cells commit to mitosis at a small cell size (Chica et al., 2016). In pancreatic beta cells, TORC1 controls cell size by regulating cell cycle progression through modulation of the synthesis and stability of cyclin D2, an important regulator of beta cell proliferation and mass buildup (Balcazar et al., 2009).

TOR complex 1 (TORC1) promotes cell growth by stimulating synthesis of the primary building blocks of macromolecules, by inhibiting autophagy and influencing cell cycle progression (De Virgilio and Loewith, 2006). TOR functions on cell physiology are achieved through crosstalk with multiple other signaling pathways. The Hippo tumor suppressor pathway regulates tissue homeostasis, cell and organ size (Pfleger, 2017). Functional connections between TOR and Hippo pathways have started to emerge: YAP and TAZ are transcriptional co-activators and represent the major effectors of the Hippo pathway (Hansen et al., 2015a; Moroishi et al., 2015). YAP and TAZ are able to activate TORC1 transcriptional induction of the high affinity leucine transporter LAT1 in HEK293 cells (Hansen et al., 2015b). In addition, YAP downregulates PTEN, a negative regulator of TOR pathway, via a posttranscriptional mechanism that involves miR-29 in the inhibition of PTEN translation (Tumaneng et al., 2012). In multicellular organisms, TOR function is regulated by growth signals through the PI3K pathway. Insulin or Insulin-like factors binding to their receptors result in insulin receptor substrate phosphorylation (IRS) and activation of PI3K. The latter converts phosphatidylinositol-4,5-phosphate (PIP2) to phosphatidylinositol-3,4,5-phosphate (PIP3) which in turn activates Akt via PDK1. Akt inhibits TSC2, thus, activating TOR (Wullschleger et al., 2006). AMP-activated protein kinase signaling (AMPK) monitors energy levels within the cell. Upon nutritional or energy stress AMPK inhibits TOR pathway (Gwinn et al., 2008; Davie et al., 2015). This relationship is conserved from yeast to man. AMPK inhibitory action on TOR is coordinated with that of the Glycogen Synthase Kinase (Gsk3) an inhibitor of the Wnt signaling pathway in metazoan and a known regulator of protein translation (Rallis et al., 2017).

Recent data indicate that TORC1 has roles in spatial cell size and growth control and organization. In budding yeast, Las24/Kog1 a TORC1 component regulates, as expected, processes that are directly related to the rapamycin-sensitive TORC1 complex. These include global protein translation as well as phosphorylation of the Ser/Thr Npr1p kinase and the Gln3p GATA transcription factor both involved in nitrogen catabolite repression (Loewith and Hall, 2011). Nevertheless, Las2/Kog1 is reported to also be implicated in the spatial arrangement of the actin cytoskeleton (Araki et al., 2005). The latter has been related so far mainly with TORC2 functions rather than TORC1. Cell growth and size in yeast have been shown to be highly dependent on actin cytoskeleton. Polarized cytoskeleton that results in morphological changes also leads to reduced cellular growth (Goranov et al., 2013). Polarization of the actin cytoskeleton inhibits TORC1 and the Iml1 complex is proved to be required for this inhibition. Interestingly, the same complex regulates the activity of TORC1 depending on the availability of nitrogen sources (Goranov et al., 2013). Both TORC1 and TORC2 protein complexes fractionate with membrane formations that are resistant to detergents and distinct from cell membrane rafts (Kunz et al., 2000; Chen and Kaiser, 2003; Wedaman et al., 2003; Aronova et al., 2007). Proteomics analysis of the TOR-containing membrane fractions was conducted revealing numerous endocytosis as well as actin cytoskeleton regulators. To validate the obtained results further genetic and biochemistry experiments were undertaken. These experiments demonstrated important numerous interactions between TORC1 and these regulators and proving the connection of TORC1 and actin cytoskeleton related functions (Aronova et al., 2007). Importantly, rapamycin is able to disrupt polarization of actin. Moreover, it can delay actin repolarization following glucose starvation and delay the localization and accumulation of Lucifer yellow -a tracer to observe kinetics of endocytosis and transportation through the vacuole membrane-within the vacuole (Aronova et al., 2007).

TOR complex 1 (TORC1) has also been implicated in special aspects of cell growth in mammals and fish. Mammalian injured mature CNS axons do not regenerate. PTEN inhibition results in TORC1-dependent, rather than TORC2, CNS axon regeneration and growth. The effect is mediated through the S6-kinase (Miao et al., 2016). Without TSC1 or TSC2 (Huang and Manning, 2008), an increase in the activity TORC1 causes regional neuronal cell growth with axons having multiple axons (Choi et al., 2008). In zebrafish, TORC1-dependent translation of ciliary precursors regulates cilia length, motility and cilia-directed flow. In particular, activation of TORC1 signaling increases ciliary length through S6 kinase 1 (S6K1)-mediated protein synthesis of cilia precursors. Inhibition of TORC1 with rapamycin, decreases ciliary length through suppression of S6K1-mediated protein translation (Yuan and Sun, 2012; Yuan S. et al., 2012). TORC1 can therefore regulate special aspects of cell growth and organization in mitotic and post mitotic cells.

## Regulation of cell size and growth aspects by TORC2

TOR complex 2 (TORC2) is reported to have opposing roles to TORC1 in fission yeast (Ikai et al., 2011). TORC2 affects cell growth and size by regulating aspects of glucose metabolism (Garcia-Martinez and Alessi, 2008; Yuan M. et al., 2012) as well as the actin cytoskeleton (Oh and Jacinto, 2011) and apoptosis (Datta et al., 1997). TORC2 activates several kinases belonging to the AGC family, such as AKT/PKB, PKC, and SGK (serum and glucocorticoid-regulated kinase) (Zoncu et al., 2011; Aimbetov et al., 2012). Interestingly, studies have shown that there is a requirement for ribosomes for TORC2 signaling. Nevertheless, protein translation is not. TORC2 protein complexes can bind with the ribosome PI3K activated by insulin is found to promotes this physical association (Zinzalla et al., 2011). Ribosomal content is a determinant of cell growth and this physical interaction could therefore serve to ensure appropriate levels of TORC2 activity in growing cells (Xie and Guan, 2011). TORC1 and TORC2 are reported to work together to regulate S6 phosphorylation in both budding and fission yeasts (Du et al., 2012; Yerlikaya et al., 2016). These findings challenge the role of ribosomal S6 protein phosphorylation in global translation. In *Drosophila*, Lst8, a component of both TORC1 and TORC2, is found to regulate cell size but not cell cycle phasing in a cell-autonomous manner through TORC2 (Wang et al., 2012). Likewise in the same animal, TORC2 regulates cell size through Myc (Kuo et al., 2015) a known regulator of organ size that induces cell competition (de la Cova et al., 2004).

Beyond its roles in spatial aspects of cell growth and size, TORC2 is also implicated in temporal aspects. Rictor null mice exhibit mild hyperglycemia and glucose intolerance that originates from the reduction in both β-cell cellular mass and multiplication (Gu et al., 2011). A known role for TORC2 is the maintenance of genome stability during S phase and its requirement for return to cell cycle progression following stress (Schonbrun et al., 2013). TORC2 has been linked to cell size upon division and the timing of mitosis: Fission yeast cells mutant for Sin1, a TORC2 component (Yang et al., 2006), divide at a longer cell size. This implies that timing of mitotic initiation is delayed (Wilkinson et al., 1999). Elongated cell morphology has also been reported in cells mutated for Tor1 (Kawai et al., 2001) and for Gad8 kinase (Matsuo et al., 2003), a known direct substrate of TORC2 related to human AKT. These data show that TORC2–Gad8 pathway promotes the initiation of mitosis. Double mutants of Tor1 and Gad8 with cdc25-22, a temperature-sensitive mutation of the Cdc25 phosphatase that activates Cdc2 kinase at G2/M (Russell and Nurse, 1986), exhibit a cell cycle arrest phenotype with highly elongated morphology. Similar phenotypes are observed when the cdc25-22 mutation is introduced to cells lacking components of the TORC2 complex, such as Sin1 and Ste20 strains (Ikeda et al., 2008). These results support the model in which TORC2 activates Gad8 which in turn advances mitosis.

Fission yeast TORC2 and Gad8 can be localized within the nucleus and bound to chromatin. Gad8 can physically interact with the MBF transcription complex which is implicated in the regulation of DNA stress response as well as the G1/S cell cycle progression. Gad8 deletion or genetic inactivation of TORC2 function (by deleting its core components) results in reduced binding of MBF to its target promoters. Moreover, MBF target genes fail to be induced in the aforementioned mutants upon DNA replication stress. Finally, the protein levels of Cdt2 and Cig2 (both of them MBF targets) are reduced when TORC2 or Gad8 function is abolished. These data highlight pivotal roles of TORC2 within the cell nucleus and reveal a TORC2-dependent mechanism in DNA replication stress response through the control of MBF transcriptional targets (Cohen et al., 2016).

Beyond its connections with cell cycle-related proteins, TORC2 is implicated in the timing of cell growth and division through interactions with the cytoskeleton. Fission yeast TORC2 regulates the timing and fidelity of cytokinesis: Disruption of TORC2 intracellular localisation or function leads in defects in cytokinetic actomyosin ring (CAR) morphology and constriction (Baker et al., 2016). Interestingly, myosin II protein Myp2 and the myosin V protein Myo51 recruit TORC2 to the CAR. TORC2 controls the fidelity of cell division and CAR stability through phosphorylation of the actin-capping protein 1 (Acp1, a regulator of cytokinesis) (Baker et al., 2016).

Roles of TORC2 in temporal aspects of cell size and growth are emerging in systems as diverse as protozoa and human cancer cells. In the protozoan parasite *Trypanosoma brucei* four TOR kinase orthologues TbTOR1, TbTOR2, TbTOR3, and TbTOR4 have been identified. TbTOR1 and TbTOR2 resemble the structure of all TOR kinases (Saldivia et al., 2013). However, TbTOR3 contains an additional PDZ domain while TbTOR4 has no FRB domain (Barquilla et al., 2008). TbTOR proteins differ in their functions, subcellular localization, and rapamycin sensitivity. Additionally, each TbTOR protein has distinct partners forming four TORC complexes (Barquilla et al., 2008, 2012; Barquilla and Navarro, 2009; de Jesus et al., 2010). TORC1 and TORC2 complexes contain KOG1/raptor and AVO3/rictor orthologues, respectively. TbTOR1 controls cell growth by regulating cell cycle, nucleolus structure, and protein synthesis, whereas TbTOR2 coordinates cell polarization and cytokinesis (Saldivia et al., 2013). Rapamycin treatment of bloodstream trypanosomes results in a profound reduction of cell proliferation. Nevertheless, this effect of rapamycin is due to exclusive action on TORC2 inhibition, with no effect on TORC1 (Barquilla et al., 2008). In tumor cells, TORC2 is required for cell cycle progression (Hietakangas and Cohen, 2008): TORC2 inhibition reduced proliferation of two cancer cell lines MCF7 and PC3. Cells lacking Rictor accumulate in G1 phase and consistently exhibit reduced levels of Cyclin D1 (Hietakangas and Cohen, 2008).

## Conclusions and future prospects

**Figure 1:**
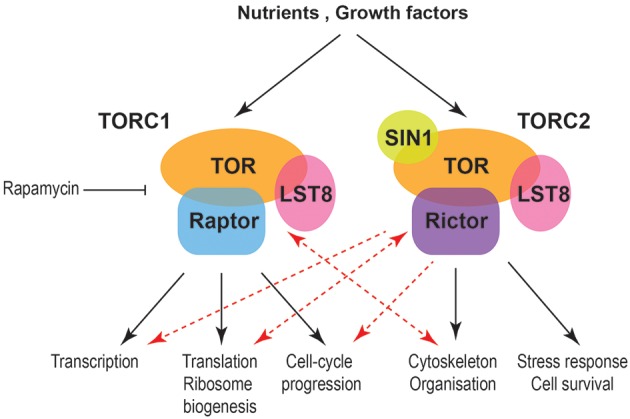
Schematic indicating processes affecting cell size and growth downstream of mammalian TORC complexes. Black arrows indicate well-established relationships between TORC1 and TORC2 with temporal and spatial aspects of cell growth, respectively. Red arrows indicate new functional connections while double red arrows indicate a cross-regulation between TORC1 and cytoskeleton as well as TORC2 and ribosomes.

Traditionally TORC1 is linked to temporal aspects of cell size and growth while TORC2 with spatial growth. Nevertheless, emerging data indicate more complex relationships (Figure 1). TORC protein complexes collect information from both intracellular and extracellular signals and are regulated in multiple levels including expression of their components and subcellular localization. Interesting directions and currently conducted work within the field include the interactions of TORC1 with cytoskeletal elements and vesicle mediated transport and their relationships with gene expression programs in cell size and growth control and during different nutritional regimes or stress. Another direction with interesting emerging results is the relationship of TORC2 with the cell cycle machinery and chromatin organization. These data will significantly enrich our knowledge on the control of cell size, growth and survival and will be pivotal for the understanding and treatment of diseases, such as cancer, diabetes and neurodegeneration.

## Author contributions

CR conceptualized the review, decided on the content, drafted the work and prepared the figures. SG provided reference searching, proofreading and editing. Both authors approve the final version of the manuscript.

### Conflict of interest statement

The authors declare that the research was conducted in the absence of any commercial or financial relationships that could be construed as a potential conflict of interest.
